# Microarray evidence of glutaminyl cyclase gene expression in melanoma: implications for tumor antigen specific immunotherapy

**DOI:** 10.1186/1479-5876-4-27

**Published:** 2006-07-04

**Authors:** John Stuart Gillis

**Affiliations:** 1 Science and Technology Studies, St. Thomas University, Fredericton, New Brunswick, Canada

## Abstract

**Background:**

In recent years encouraging progress has been made in developing vaccine treatments for cancer, particularly with melanoma. However, the overall rate of clinically significant results has remained low. The present research used microarray datasets from previous investigations to examine gene expression patterns in cancer cell lines with the goal of better understanding the tumor microenvironment.

**Methods:**

Principal Components Analyses with Promax rotational transformations were carried out with 90 cancer cell lines from 3 microarray datasets, which had been made available on the internet as supplementary information from prior publications.

**Results:**

In each of the analyses a well defined melanoma component was identified that contained a gene coding for the enzyme, glutaminyl cyclase, which was as highly expressed as genes from a variety of well established biomarkers for melanoma, such as MAGE-3 and MART-1, which have frequently been used in clinical trials of melanoma vaccines.

**Conclusion:**

Since glutaminyl cyclase converts glutamine and glutamic acid into a pyroglutamic form, it may interfere with the tumor destructive process of vaccines using peptides having glutamine or glutamic acid at their N-terminals. Finding ways of inhibiting the activity of glutaminyl cyclase in the tumor microenvironment may help to increase the effectiveness of some melanoma vaccines.

## Background

In recent decades considerable progress has been made in developing methods which use the immune system to treat cancer. One particularly noteworthy series of events was the discovery of the first genes encoding human tumor antigens recognized by cytolytic T lymphocytes (CTLs) [1-3]. Since then, dozens of other antigen genes have been identified [4], many of which contain several short sequences of DNA, coding for peptides called "epitopes." When epitopes are bound by members of the human leukocyte antigen (HLA) family, presented on the tumor cell surface, and recognized by CTLs; destruction of the tumor cell tends to occur. Scientists have become so adept at using such methods for reproducibly destroying tumor cells in the laboratory that many small scale clinical vaccine trials have been carried out [5]. Unfortunately, although there have been reports of some dramatic remissions, the overall degree of successful therapy has remained low [6].

Explanations for the difficulty in translating findings from the laboratory to the clinic in the field of tumor antigen (TA) specific immunotherapy have focussed upon a variety of factors, one of which has been the importance of better understanding the tumor microenvironment [7]. In this regard, it has been suggested that breakthroughs in the development of microarray technology may be able to help overcome tumor resistance to effective vaccine responses [5,8].

The present report is of a retrospective microarray datamining study looking for information relevant to the tumor microenvironment in TA specific immunotherapy.

## Methods

The first dataset analysed in the current research was published in a study by Ross *et al *[9] using the National Cancer Institute (NCI) group of 60 cancer cell lines from 9 different tissues of origin: melanoma, colon, renal, breast, CNS, leukemia, prostate, lung, and ovarian. For each of the NCI60 cell lines, expression levels of approximately 8000 genes were obtained from a microarray upon which 9706 cDNAs had been physically deposited. This dataset is available at the **Genomics and Bioinformatics Group Microarray Datasets **website [10].

The second dataset was collected by Staunton *et al *[11] using the NCI60 cell lines and an Affymetrix microarray, upon which short (25-mer) oglionucleoties had been directly synthesized. Using this form of microarray platform 7130 gene expression levels were measured. The raw data has been deposited at the **Genomics and Bioinformatics Group Microarray Datasets **website [12].

The third dataset used here was published in conjunction with a paper by Györffy *et al *[13] using an Affymetrix HGU133 microarray chip containing 42297 estimates of gene expression levels. This study utilized 30 cancer cell lines from 9 different types of human tissue: breast, liver, lung, melanoma, ovarian, pancreas, colon, gastric and prostate. The microarray raw dataset was made available in supplementary Table 1 published with the article.

The primary method used in the present research was the multivariate statistical technique of principal components analysis (PCA), previously utilized with microarray data in a study by Crescenzi and Giuliani [14]. However, unlike the Crescenzi and Giuliani research, in which cDNAs with values for all 60 cell lines were used, the present PCA was carried out after replacing any missing values with the mean for each cell line. This meant that, instead of using 1416 cDNAs, there were 9706 values included in the PCA. Another important difference was that, following the main principal components extraction, Promax rotational transformations were performed [15]. These alternative, complementary procedures were undertaken to see if PCA could produce results in addition to the interesting findings obtained in earlier research [14].

Additional analyses were carried out using: (a) Pearson product-moment correlation coefficients, (b) linear discriminant analysis, and (c) unpaired two-tailed Student *t *tests for the difference between means from independent samples. Prior to each *t *test, *F *ratios were calculated to ascertain whether to assume equality of variances.

All analyses were performed with SPSS for Windows XP (Version 12.0) using default values for each procedure unless otherwise indicated. Prior to the PCA, the NCI60 Affymetrix microarray expression estimates were converted to standardized z-scores.

## Results

### Ross *et al *cDNA microaarray data

**Figure 1:**
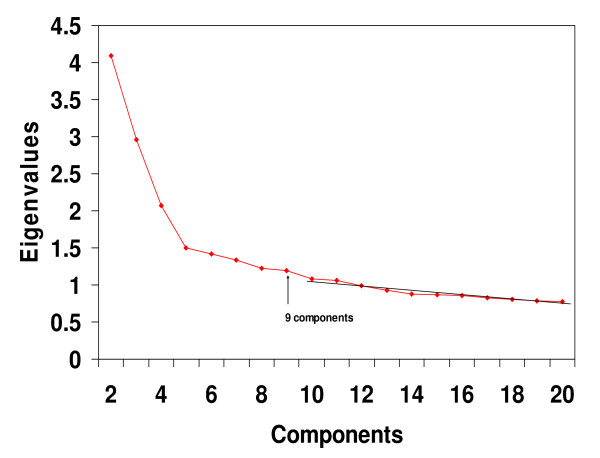
**Scree test for the Ross *et al *dataset**. Eigenvalues from the 60 × 60 cell line correlation matrix showed that, beginning with the break in the plot after component 9, a straight scree line could be fitted to the remaining values. Such a finding suggests that the eigenvalues after component 9 represent only error variance. The term "scree" was borrowed from geology where it refers to debris (i.e. error) that has fallen to the base of a mountain. Note that for clarity of presentation, the first eigenvalue of 13.787 was not plotted.

Pearson product-moment correlation coefficients were calculated between the cell lines, and then eigenvalues for the 60 × 60 correlation matrix were calculated. A scree test [14,16,17], presented in Figure 1, was performed using the eigenvalues plot. The scree results indicated that it would be appropriate to extract 9 components. Following extraction of 9 principal components, Promax rotations were carried out. As may be seen in the Promax component pattern loading values presented in Table 1 (see Additional file 1), there were 68% of the loadings within the +/-.10 hyperplane, which indicated that a high degree of simple structure had been achieved [17-19]. In addition, it may be seen in Table 1 (see Additional file 1) that Component #1 consisted of the melanoma cell lines: MALME3M, SLMEL2, SKMEL5, SKMEL28, M14, UACC62 and UACC257 together with MDAMB435 and MDAN. This group of cell lines is identical with the Melanoma Cluster found previously by hierarchical clustering in two separate research reports (see Figure 1 of Ross et al [9] and cluster 3 in Figure 3 of Crescenzi and Giuliani [14]).

**Figure 2:**
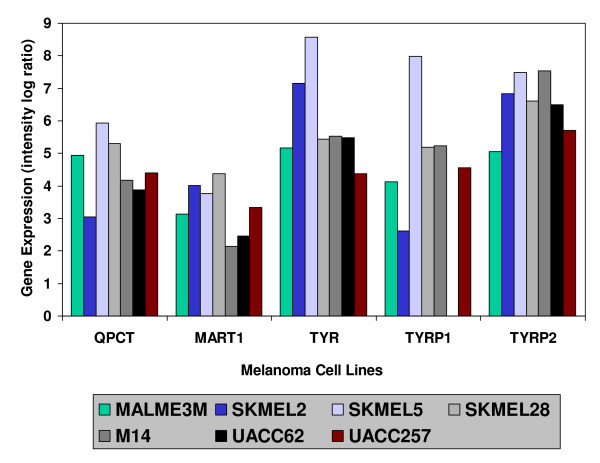
**Highly expressed genes within the Ross *et al *Melanoma Component**. Gene expression levels of 5 genes within each of the 7 cell lines in the Ross *et al *cDNA Melanoma Component. Measurements were log ratios between the gene expression level of each cell line and a reference sample of 12 of the 60 tumor cell lines. Further details may be found in the original Ross *et al *publication. The means for each gene for the non-melanoma cell lines (followed by the Student *t test *significances for the mean difference between melanoma and non-melanoma cell lines) were as follows: QPCT -.514 (p < .001), MART1 -.055 (p = .001), TYR -.031 (P < .001), TYRP1 -.449 (P < .001), and TYRP2 -.041 (p < .001). It may be seen that the means of the non-melanoma cell lines were all very close to zero, and therefore were not plotted simply for greater clarity of presentation.

**Figure 3:**
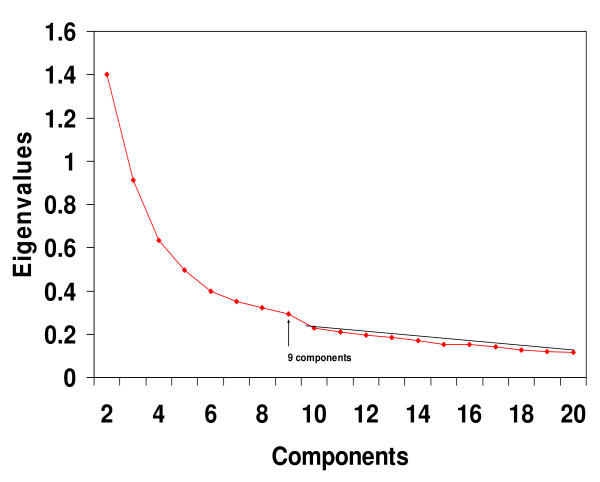
**Scree test for the Staunton *et al *dataset**. Eigenvalues from the 60 × 60 cell line correlation matrix showed that, beginning with the break in the plot after component 9, a straight scree line could be fitted to the remaining values. Such a finding suggests that the eigenvalues after component 9 represent only error variance. Note that for clarity of presentation, the first eigenvalue of 51.392 was not plotted.

It should be noted that there is evidence the cell lines, MDAMB435 and MDAN (derived from the same patient), originally believed to be of breast cancer in origin, may have resulted from melanoma metastasis [9]. At the same time, the allegedly melanoma cell line, LOXIMVI, which had previously fallen outside of the Melanoma Cluster [9,14], also did not appear in the present Melanoma Component. Because of these questions about the cancer type of these three cell lines, as a conservative measure, their scores were not included in further statistical analyses.

In order to identify the most highly expressed genes in the cell lines comprising the Melanoma Component, regression scores were calculated for each of the 9706 cDNAs. The 26 highest scoring genes of the Melanoma Component (together with the component regression score for each gene) are listed in Table 2 (see Additional file 2), followed by the 26 genes found by Ross *et al *within their Melanoma Cluster. In Table 2 (see Additional file 2) it may be seen that 14 of the genes identified by Ross *et al *were among the highest scoring in the present Melanoma Component. From the 14 genes falling in both the Ross *et al *and the present melanoma group, several genes were chosen for presentation in Figure 2, these genes were: Melanoma Antigen recognized by T-cells (MART1), Tyrosinase (TYR), Tyrosinase Related Protein 1 (TYRP1) and Tyrosinase Related Protein 2 (TYRP2). These four genes were selected because they are well known biomarkers for melanoma and have been utilized as epitopes in tumor immunotherapy [5]. Also presented in Figure 2 was the gene, Glutaminyl Cyclase (QPCT). The reason for selecting QPCT was that, as may be seen in later subsections, it was found to be highly expressed in two other microarray datasets.

Next, to determine how a combination (and not only a gene-by-gene analysis) of the selected genes perform on the melanoma identification, a linear discriminant analysis [20] was performed using MART1, TYR, TYRP1, TYRP2 and QPCT as independent variables and melanoma vs non-melanoma PCA scores as the grouping variable. It was found that the overall gene combination was related to the melanoma distinction (Wilks Lambda = .057, p < .001) with the standardized canonical discriminant function coefficients being MART1 = .267, TYR = .420, TYRP1 = .361, TYRP2 = .595, and QPCT = .808.

### Staunton *et al *Affymetrix microaarray data

As with the cDNA analysis, a scree test with the eigenvalues from the 60 × 60 NCI Affymetrix correlation matrix indicated that 9 components should be extracted (Figure 3). PCA was performed on the NCI60 Affymetrix gene expression estimates. The PCA produced results that were highly similar to those obtained with the cDNA values analysed in the original Ross *et al *study [9]. It was found that PROMAX rotations (with the maximum iterations for convergence parameter set at 50 instead of the default value of 25) resulted in a readily identifiable Melanoma Component (component pattern loading values are presented in Table 3 – see Additional file 3) made up of the cell lines, MALME3M, SLMEL2, SKMEL5, SKMEL28, M14, UACC62 and UACC257.

**Figure 4:**
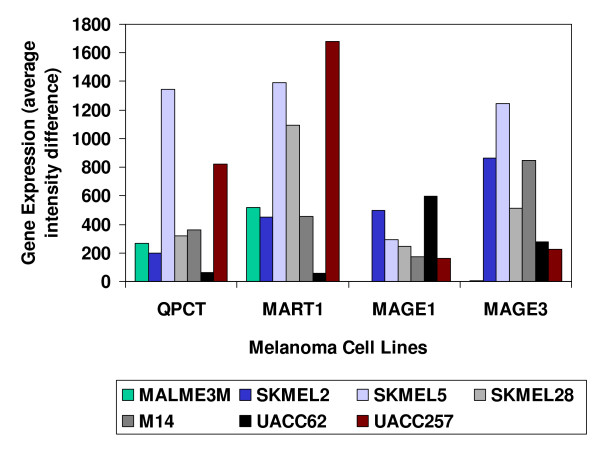
**Highly expressed genes within the Staunton *et al *Melanoma Component**. Gene expression levels of 4 genes within each of the 7 cell lines in the Staunton *et al *Affymetrix Melanoma Component together with the mean expression levels for the combined non-melanoma cell lines. The expression values were average intensity difference units determined with Affymetrix GENECHIP software that assigned a value of 100 to all expression measurements of less than 100 units. Further details may be found in the Staunton *et al *publication. The Student *t test *significance levels for each gene, calculated between the melanoma and the non-melanoma cell lines, were as follows: QPCT (p = .033), MART1 (p = .012), MAGE1 (p = .019) and MAGE3 (p = .024). Note that for clarity of presentation, this figure used expression average intensity difference units, as they were prior to conversion to the standardized z-scores that were used in the PCA and *t *test calculations.

When component scores were calculated for each of the genes on the microarray, the enzyme QPCT again was found to be as highly expressed (see Figure 4) as some well known proteins used in melanoma immunotherapy [5]: MART1, Melanoma Antigen 1 (MAGE1) and Melanoma Antigen 3 (MAGE3).

### Combined Ross *et al *and Staunton *et al *data analysis

In order to show the effective consistence of the Staunton *et al *Melanoma Component (shown in Table 3 – see Additional file 3) with the previously identified Melanoma Component from the Ross *et al *data (depicted in Table 1 – see Additional file 1), a Pearson Correlation Coefficient was calculated between the loading values. The obtained value of .905 (p < .001) supported a hypothesis of identity between these component patterns.

### Györffy *et al *Affymetrix microarray data

**Figure 5:**
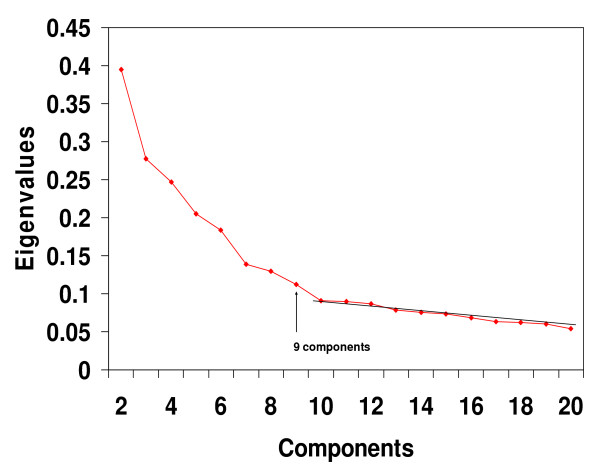
**Scree test for the Györffy *et al *Affymetrix dataset**. Eigenvalues from the 30 × 30 cell line correlation matrix showed that, beginning with the break in the plot after component 9, a straight scree line could be fitted to the remaining values. Such a finding suggests that the eigenvalues after component 9 represent only error variance. Note that for clarity of presentation, the first eigenvalue of 27.020 was not plotted.

As with the other 2 analyses, PCA results with the Affymetrix HGU133 microarray chip produced results that matched well with previous findings. A scree test indicated that 9 components should be extracted (Figure 5). Following components extraction and Promax rotations, one of the 9 components clearly corresponded to the Melanoma Cluster found in the original Györffy *et al *study [13]. The Melanoma Component (component pattern loading values are presented in Table 4 – see Additional file 4) was composed primarily of the melanoma cell lines ME43, MEWO, A375, SKMEL13 and SKMEL19. One lung cell line, COLO699, loaded significantly on the Melanoma Component, and one melanoma cell line, C8161, did not load. However, both of these findings also occurred in the hierarchical cluster analysis of the Györffy *et al *study (see their Figure 5) [13].

**Figure 6:**
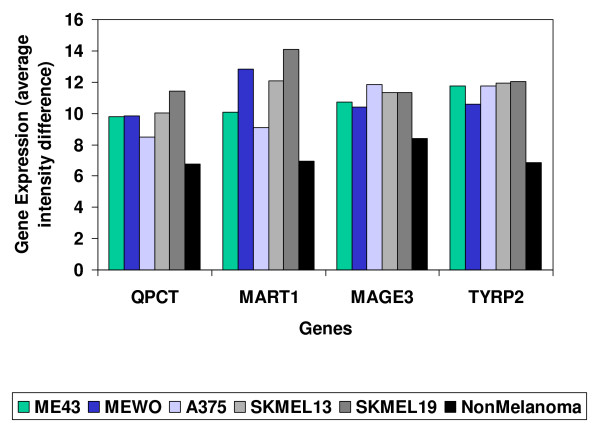
**Highly expressed genes within the Györffy *et al *Melanoma Component**. Gene expression levels of 4 genes within each of the 5 cell lines in the Györffy *et al *Melanoma Component together with the mean expression levels for the combined non-melanoma cell lines. The expression values were average intensity difference units determined with Affymetrix MAS 5.0 software. Further details may be found in the Györffy *et al *publication. The Student *t test *significance levels for each gene, calculated between the melanoma and the non-melanoma cell lines, were as follows: QPCT (p < .001), MART1 (p = .010), MAGE3 (p < .001) and TYRP2 (p < .001).

Interestingly, the QPCT enzyme again was expressed at a level similar to well established antigens used in melanoma vaccines [5]: MART1, MAGE3 and TYRP2 (Figure 6).

## Discussion

Prior to the present findings there had been several indications that the gene coding for glutaminyl cyclase tends to be highly expressed in melanoma. In an exploratory microarray study aimed at identifying potential immunological targets in tumor microenvirnoments, Wang *et al *[21] published an additional file listing 2044 clones which appeared to be upregulated in metastatic cutaneous melanoma. Included in this list of cDNA clones was glutaminyl cyclase.

In another study, employing the high throughput method of Serial Analysis of Gene Expression to survey the gene expression patterns of melanoma tumor samples, Weeraratna *et al *[22] identified glutaminyl cyclase as being among those most abundantly expressed [see their Figure 1 (c)].

Consequently, when combined with the present findings, there is now evidence from a variety of sources supporting the notion that glutaminyl cyclase tends to be highly expressed in melanoma.

Evidence about the potential significance of this finding for immunological treatment of cancer comes from several sources. Firstly, it is interesting to note that several highly researched epitopes, such as MAGE3 (EVDPIGHLY) and the synthetic MART1 (ELAGIGILTV), which are among those having produced the most clinically interesting results [23-26], have an E at their N-terminal. Secondly, it has been shown that the main function of glutaminyl cyclase is to accelerate the transformation of glutamine (Q) or glutamic acid (E) into pyroglutamic acid (pE) [27,28]. Thirdly, when either Q or E occur at the N terminal of an epitope, their cyclic transformation to pE has been shown to decrease the affinity with which the peptide will bind with HLA molecules [29,30]. Hence, high levels of glutaminyl cyclase within melanoma cells may interfere with the avidity with which T cells recognize and destroy tumors.

The plausibility of such a scenario is increased by knowledge that the generation of antigenic peptides is a multistep process. Part of this process takes place in the endoplasmic reticulum (ER), and the glutaminyl cyclase gene codes for an ER targeting signal [31].

Should laboratory research confirm the present microarray-derived indication of QPCT activity within the melanoma tumor microenvironment, attention may be focused upon finding ways to overcome QPCT interference. In this regard it is interesting to note that potent new inhibitors have been discovered recently for QPCT [32]. However, application of such inhibitors in a clinical setting is likely to present difficulties, because QPCT plays a significant role in many normal physiological processes, such as influencing the bioactivity of hormones like gonadotropin-releasing hormone and thyrotropin-releasing hormone [33]. It is also noteworthy that QPCT may be essential for the activity of monocyte chemotactic protein 2 (MCF-2), since MCF-2 is practically inactive without a pE at its N-terminal [34]. Perhaps the development of tissue specific inhibitors could help to overcome problems due to the widespread activity of glutaminyl cyclase [35].

Another possible difficulty is that there is some evidence that glutaminyl cyclase, like MART-1, TYR, and TYRP2, may be highly expressed in normal melanocytes as well as in melanoma (see Table 1 of Dooley *et al *[36]). Further research is needed to find ways to overcome such potential problems in translating results from the present basic research into clinical immunotherapy.

## Competing interests

The author(s) declare that they have no competing interests.

## Supplementary Material

Additional file 1 Table 1: Promax component pattern loading values for the first 4 components of the Ross *et al *dataset. Click here for file

Additional file 2 Table 2: Biomarker genes in (a) the present melanoma component and (b) the original Ross *et al *cluster. Click here for file

Additional file 3 Table 3: Promax melanoma component pattern loadings for the Staunton *et al *dataset. Click here for file

Additional file 4 Table 4: Promax melanoma component pattern loading values for the Györffy *et al *dataset. Click here for file
