# Supplementary material for: Microarray evidence of glutaminyl cyclase gene expression in melanoma: implications for tumor antigen specific immunotherapy
Source: J Transl Med. 2006 Jul 4;4:27. doi: 10.1186/1479-5876-4-27 (PMC1557589; doi:10.1186/1479-5876-4-27)
Supplement: Additional file 1 — Table 1: Promax component pattern loading values for the first 4 components of the Ross et al dataset. [file 1479-5876-4-27-S1.pdf]

Table 1: Promax component pattern loading values for the first 4 components of the Ross *et al* dataset.

		Component number			
**Cell line name**	Cancer type	**1**	**2**	**3**	**4**
**MEMALME3M**	**Melanoma**	*.739**	.032	−.012	−.123
**MESKMEL2**	**Melanoma**	*.647**	.015	.059	.045
**MESKMEL5**	**Melanoma**	*.681**	.027	−.010	.042
**MESKMEL28**	**Melanoma**	*.882**	−.013	.040	−.105
**MEM14**	**Melanoma**	*.743**	.026	−.020	−.038
**MEUACC62**	**Melanoma**	*.748**	.010	.017	−.014
**MEUACC257**	**Melanoma**	*.760**	−.057	−.029	.043
**COHT29**	**Colon**	−.111	*.692*	.080	.107
**COHCC2998**	**Colon**	.042	*.803*	.045	.022
**COHCT116**	**Colon**	.068	*.455*	−.068	−.020
**COSW620**	**Colon**	.045	*.579*	−.100	.172
**COHCT15**	**Colon**	−.019	*.637*	−.054	.096
**COKM12**	**Colon**	.012	*.881*	−.036	−.070
**COCOLO205**	**Colon**	.052	*.881*	−.078	.051
**REUO31**	**Kidney**	.003	.085	*.546*	−.076
**REA498**	**Kidney**	−.014	−.007	*.645*	.004
**RECAKI1**	**Kidney**	−.030	−.092	*.646*	.072
**RERXF393**	**Kidney**	.037	−.165	*.710*	.072
**RE7860**	**Kidney**	−.024	−.013	*.668*	.106
**REACHN**	**Kidney**	.045	.052	*.579*	−.066
**RETK10**	**Kidney**	.029	.001	*.632*	.018

LECCRFCEM	Leukemia	-.053	.016	.035	*.816*
LEK562	Leukemia	-.002	-.071	-.090	*.685*
LEMOLT4	Leukemia	-.035	.062	-.005	*.806*
LESR	Leukemia	-.016	.023	.129	*.596*
LEHL60	Leukemia	-.018	.012	.019	*.832*
LERPMI8226	Leukemia	.085	.022	.134	*.612*
CNSSNB19	Brain	.028	-.083	-.046	.001
CNSSNB75	Brain	.241	-.099	.005	-.088
CNSU251	Brain	-.060	-.018	-.077	-.043
CNSSF268	Brain	-.034	-.125	-.093	.036
CNSSF295	Brain	-.015	.006	.083	.078
CNSSF539	Brain	.027	.065	.019	.037
LCNCIH23	Lung	.026	-.003	-.025	.166
LCNCIH522	Lung	.027	-.177	-.156	.163
LCA549ATCC	Lung	-.033	.049	.172	-.101
LCEKVX	Lung	-.060	.054	.092	-.148
LCNCIH322M	Lung	.012	.573	-.001	-.234
LCNCIH460	Lung	.072	-.021	.117	.025
LCHOP62	Lung	-.078	.022	.081	.035
LCHOP92	Lung	.041	.195	.128	-.024
LCNCIH226	Lung	-.120	.167	.113	-.159
OVOVCAR3	Ovarian	.004	-.032	-.055	-.057
OVOVCAR4	Ovarian	.017	-.125	.121	-.059
OVOVCAR8	Ovarian	.002	-.117	.032	-.072
OVIGROV1	Ovarian	.044	.065	.235	.067
OVSKOV3	Ovarian	-.050	.138	.271	-.056
OVOVCAR5	Ovarian	.018	.470	.123	-.120

BRMCF7	Breast	-.007	.259	-.195	.162
BRMCF7ADFRES	Breast	.061	-.109	.013	-.045
BRMDAMB231	Breast	-.010	.169	.074	.041
BRHS578T	Breast	.070	-.078	.004	-.140
BRBT549	Breast	-.055	-.009	.033	.071
BRT47D	Breast	.035	.279	-.076	.090
PRPC3	Prostate	.005	.195	-.010	.132
PRDU145	Prostate	-.032	.187	.163	-.005
RESN12C	Kidney	-.020	-.070	-.029	.069
BRMDAMB435	Melanoma/Breast?	*.687**	.002	-.048	.052
BRMDAN	Melanoma/Breast?	*.736**	.054	.006	.105
MELOXIMVI	Melanoma?	.146	-.027	.096	.221

* indicates a loading that came from a cell line, which was found to be a member of the Melanoma Cluster, in both the Ross *et al* and Crescenzi & Giuliani studies.
