# Supplementary material for: Microarray evidence of glutaminyl cyclase gene expression in melanoma: implications for tumor antigen specific immunotherapy
Source: J Transl Med. 2006 Jul 4;4:27. doi: 10.1186/1479-5876-4-27 (PMC1557589; doi:10.1186/1479-5876-4-27)
Supplement: Additional file 2 — Table 2: Biomarker genes in (a) the present melanoma component and (b) the original Ross et al cluster. [file 1479-5876-4-27-S2.pdf]

Table 2: Biomarker genes in (a) the present melanoma component and (b) the original Ross *et al* cluster.

(a) Highest scoring genes from the present study melanoma component.

Gene	Component Regression Score
TYRP2 DCT DOPACHROME TAUTOMERASE *	8.11
TYR TYROSINASE *	7.00
SKELETAL MUSCLE-SPECIFIC CALPAIN	6.64
S-100 PROTEIN, BETA *	5.92
TYRP1 TYROSINASE-RELATED PROTEIN 1*	5.59
DDS1BETA PROTEIN	5.56
ACP5 ACID PHOSPHATASE TYPE 5*	5.32
QPCT GLUTAMINYL CYCLASE	5.30
ENDOTHELIN RECEPTOR TYPE B	4.95
EDRA3 CAGH3 *	4.71
BCHE BUTYRYLCHOLINESTERASE*	4.51
HTMART MONO-ADP-RIBOSYLTRANSFERASE	4.23
GALECTIN 3	3.90
RXRG RETINOID X RECEPTOR GAMMA *	3.86
MBP MYELIN BASIC PROTEIN*	3.76
CLASS II HISTOCOMPATIBILITY ANTIGEN, M	3.70
BILIARY GLYCOPROTEIN	3.58
DGCR6 PROTEIN	3.56
SIAT8A ALPHA2,8-SIALYLTRANSFERASE *	3.48
A2M ALPHA-2-MACROGLOBULIN*	3.47
MART-1*	3.45
MUTYH MUTY HOMOLOG *	3.42
SCRAPIE RESPONSIVE PROTEIN 1*	3.22
FARNESYLTRANSFERASE	3.18
CLATHRIN COAT ASSEMBLY PROTEIN AP19	3.18
CMP-N-ACETYLNEURAMINATE	3.10

*** Genes also found in Ross et al original cluster listed below**

(b) Genes included in the Ross *et al* original melanoma cluster

CMOAT
G2PROTEIN
PTPRZ1 TYROSINE PHOSPHATASE ZETA
MBP MYELIN BASIC PROTEIN*
BCHE BUTYRYLCHOLINESTERASE*

SCRAPIE RESPONSIVE PROTEIN 1*
ART3 MONO-ADP-RIBOSYLTRANSFERASE
SIAT6 ALPHA2,3-SIALYLTRANSFERASE
ACP5 ACID PHOSPHATASE TYPE 5*
RXRG RETINOID X RECEPTOR GAMMA*
TYRP2 DCT DOPACHROME TAUTOMERASE*
TYR TYROSINASE*
MUTYH MUTY HOMOLOG*
S100B S-100 BETA*
EDNRB ENDOTHELIN RECEPTOR TYPE B
TYRP1 TYROSINASE-RELATED PROTEIN 1*
SIAT8A ALPHA2,8-SIALYLTRANSFERASE*
MELANOMA ANTIGEN RECOGNIZED BY T-CELLS (MART-1)*
GM6B
PDNP2 NUCEOTIDE PYROPHOSPHATASE 2
EDRA3 CAGH3*
CPB2 PLASMA CARBOXYPEPTIDASE B
LGALS3BP GALECTIN-6 BINDING PROTEIN
A2M ALPHA-2-MACROGLOBULIN*
AACT ALPHA-1-ANTICHYMOTRYPSIN
LAMA4 LAMININ ALPHA 4

*** Genes also found in present Melanoma Cluster**
