# Supplementary material for: Microarray evidence of glutaminyl cyclase gene expression in melanoma: implications for tumor antigen specific immunotherapy
Source: J Transl Med. 2006 Jul 4;4:27. doi: 10.1186/1479-5876-4-27 (PMC1557589; doi:10.1186/1479-5876-4-27)
Supplement: Additional file 3 — Table 3: Promax melanoma component pattern loadings for the Staunton et al dataset. [file 1479-5876-4-27-S3.pdf]

Table 3: Promax melanoma component pattern loadings for the Staunton *et al* dataset.

Cell line name	Cancer type	Loading
MELAN_MALME3M	Melanoma	*.629*
MELAN_SKMEL2	Melanoma	*.413*
MELAN_SKMEL5	Melanoma	*.231*
MELAN_SKMEL28	Melanoma	*.603*
MELAN_M14	Melanoma	*.367*
MELAN_UACC62	Melanoma	*.472*
MELAN_UACC257	Melanoma	*.599*
COLON_HT29	Colon	-.195
COLON_HCC2998	Colon	-.086
COLON_HCT116	Colon	-.023
COLON_SW620	Colon	-.091
COLON_HCT15	Colon	-.085
COLON_KM12	Colon	-.074
COLON_COLO205	Colon	-.141
RENAL_UO31	Kidney	-.025
RENAL_A498	Kidney	-.046
RENAL_CAKI1	Kidney	-.219
RENAL_RXF393	Kidney	-.044
RENAL_7860	Kidney	-.126
RENAL_ACHN	Kidney	.030
RENAL_TK10	Kidney	-.129
LEUK_CCRFCEM	Leukemia	-.131
LEUK_K562	Leukemia	-.011
LEUK_MOLT4	Leukemia	-.094
LEUK_SRR	Leukemia	.086
LEUK_HL60	Leukemia	-.116
LEUK_RPMI8266	Leukemia	.020
CNS_SNB19	Brain	-.076
CNS_SNB75	Brain	-.059
CNS_UU251	Brain	-.104
CNS_SF268	Brain	.089
CNS_SF295	Brain	-.052
CNS_SF539	Brain	-.021

NSCLC_NCIH23	Lung	-.005
NSCLC_NCIH522	Lung	-.064
NSCLC_A549ATCC	Lung	-.025
NSCLC_EKVX	Lung	.090
NSCLC_NCIH332M	Lung	-.113
NSCLC_H460	Lung	-.019
NSCLC_HOP62	Lung	-.217
NSCLC_HOP92	Lung	-.009
NSCLC_NCIH226	Lung	-.053
OVAR_OVCAR3	Ovarian	-.051
OVAR_OVCAR4	Ovarian	-.122
OVAR_OVCAR8	Ovarian	-.085
OVAR_IGROV1	Ovarian	-.090
OVAR_SKOV3	Ovarian	-.194
OVAR_OVCAR5	Ovarian	-.064
BREAST_MCF7	Breast	-.095
BREAST_MCF7ADRFRES	Breast	-.174
BREAST_MDAMB231	Breast	-.026
BREAST_HS578T	Breast	-.078
BREAST_BT549	Breast	.032
BREAST_T47D	Breast	-.120
PROSTATE_PC3	Prostate	-.072
PROSTATE_DU145	Prostate	-.226
RENAL_SN12C	Kidney	-.213
