# Supplementary material for: Microarray evidence of glutaminyl cyclase gene expression in melanoma: implications for tumor antigen specific immunotherapy
Source: J Transl Med. 2006 Jul 4;4:27. doi: 10.1186/1479-5876-4-27 (PMC1557589; doi:10.1186/1479-5876-4-27)
Supplement: Additional file 4 — Table 4: Promax melanoma component pattern loading values for the Györffy et al dataset. [file 1479-5876-4-27-S4.pdf]

Table 4: Promax melanoma component pattern loading values for the Györffy *et al* dataset.

Cell Line Name	Cancer type	Loading
ME43	Melanoma	*.740*
MEWO	Melanoma	*.629*
SKMEL13	Melanoma	*.770*
SKMEL19	Melanoma	*.781*
A375	Melanoma	*.758*
COLO699	Lung	*.556*
C8161	Melanoma	.076
BT20	Breast	.130
R103	Breast	.169
R193	Breast	.100
SKBR3	Breast	.192
MDA231	Breast	.160
ES2	Ovarian	.214
FUOV1	Ovarian	.093
OAW42	Ovarian	.154
OVKAR	Ovarian	.120
SKOV3	Ovarian	.137
HEP3B	Liver	.096
SNU182	Liver	.079
SNU423	Liver	.168
SNU449	Liver	.075
SNU475	Liver	.215
CX2	Colon	.148
HRT18	Colon	.078
HT29	Colon	.093
DU145	Prostate	.153
SW13	Prostate	.108
181P	Pancreas	.093
257P	Gastric	.175
DV90	Lung	.162
